# Integer programming model extensions for a multi-stage nurse rostering problem

**DOI:** 10.1007/s10479-017-2623-z

**Published:** 2017-09-01

**Authors:** Florian Mischek, Nysret Musliu

**Affiliations:** Database and Artificial Intelligence Group, Vienna University of Technology, Vienna, Austria

**Keywords:** Nurse rostering, INRC-II, Integer programming

## Abstract

In the variant of the well studied nurse rostering problem proposed in the Second International Nurse Rostering Competition, multiple stages have to be solved sequentially which are dependent on each other. We propose an integer programming model for this problem and show that a set of newly developed extensions in the form of additional constraints to deal with the incomplete information can significantly improve the quality of the generated solutions. We compare our solution approaches with the results obtained in the competition and show that the extended model achieves results competitive with the competition finalists.

## Introduction

The automated generation of high quality staff schedules, in particular for hospitals, has been an important problem for over 40 years. Multiple variants and solution approaches exist [to be found e.g. in surveys by Ernst et al. (2004) and den Bergh et al. (2013)]. A survey focused on rostering problems in hospitals specifically was published by Burke et al. (2004).

Ceschia et al. (2015) proposed a variant of the Nurse Rostering Problem for the Second International Nurse Rostering Competition (INRC-II). In contrast to previous problem variants, a multi-stage formulation is used, where solutions for individual weeks have to be produced by the solver sequentially, without information about the requirements of later weeks. Salassa and Vanden Berghe (2012) denoted such a setting as a *stepping horizon* approach.

This multi-stage setting poses two unique challenges for solvers: The dependencies between weeks make it necessary to take the solutions of previous weeks into account during the evaluation of the quality of a schedule. Publications treating this issue are by Glass and Knight (2010), Salassa and Vanden Berghe (2012) and Smet et al. (2016), who developed and formalized various strategies to consistently include the results of previous stages in the evaluation of the current schedule. Their findings have largely been included in the rules for constraint evaluation of the INRC-II.

Further, and not explored in the previously mentioned papers, is the fact that due to the incomplete information during all weeks but the last, the generated solution can no longer be guaranteed to be optimal even if each week is solved to optimality. More so, a naive model that is not adapted to this setting will produce imbalanced schedules that incur large penalties in later weeks as options are restricted excessively by the solutions of the previous weeks. It is therefore necessary to allow for the existence of future scheduling periods already during the solution of the current stage. While e.g. Salassa and Vanden Berghe (2012) successfully improve their results by regarding the solutions of past stages, they do not include any special mechanisms to account for the upcoming future stages. To the best of our knowledge, the same holds for the other works dealing with such multi-stage settings.

There have been 15 submissions to the INRC-II, with seven of these advancing to the final round. The results for all participants are available on the competition website,^1^ while information about some solution approaches (Dang et al. 2016; Kheiri et al. 2016; Jin et al. 2016; Römer and Mellouli 2016) has been published as extended abstracts in the proceedings of PATAT2016. In particular, the winners of the competition used integer programming (IP) with a network-flow-based formulation (Römer and Mellouli 2016). One other publication dealing with this problem is by Santos et al. (2015), who used a weighted constraint satisfaction approach.

Several IP formulations have been previously used for nurse rostering problems, including the problem proposed in the First International Nurse Rostering Competition (INRC2010) (Haspeslagh et al. 2014). For this (single-stage) problem, Santos et al. (2016) provided an IP formulation and proposed techniques to improve the performance of IP solvers based on this formulation, by providing good dual and primal bounds. Valouxis et al. (2012) proposed a two phase approach for the INRC2010 problem. Integer programming formulations were proposed in both phases to assign nurses to working days and to shift types. A branch and price algorithm has been proposed for solving INRC2010 instances in Burke and Curtois (2014). In this competition, also several heuristic and hybrid algorithms have been applied to the instances. We refer the reader to Haspeslagh et al. (2014) for a comparison of different approaches. IP has also been used for other nurse rostering problems [e.g. Burke et al. (2010), Brucker et al. (2011)].

In this article, we investigate solving a new nurse rostering problem (proposed in the INRC-II) by Integer Programming. As in this problem a multi-stage formulation is used, the application of previous IP approaches is not sufficient to obtain good solutions that take into consideration future scheduling periods. Therefore, novel formulations are needed to cope with this new problem. We first propose a basic IP formulation (Sect. 3) for the INRC-II problem (as defined in Sect. 2). Our main contribution is the extension of this model with new additional constraints to account for the multi-stage setting (see Sect. 4). To the best of our knowledge this extension presents an original contribution to the literature.

We evaluate our formulations in Sect. 5, using the instances provided for the INRC-II and show that the additional constraints significantly improve the quality of the generated solutions. We also compare our model with the results of the finalists in the INRC-II. In this comparison, the results of the extended (IP) model are competitive (slightly better than the median).

This paper is an extension of work previously published in the proceedings of the 2016 conference on the *Practice and Theory of Automated Timetabling* Mischek and Musliu (2016), which was further based on the first author’s master’s thesis [see Mischek (2016)].

## Problem definition

In this section, we give a short overview of the problem used in the INRC-II. A detailed description of the problem structure and all constraints can be found in the competition rules (Ceschia et al. 2015).

Instances are of either 4 or 8 weeks duration. For each week, a schedule has to be found by the solver, using only the information provided in a global *scenario* file, containing information about the nurses and their contracts, *week data* about the requirements of the current week and a *history* with data concerning the last assignments of the previous weeks and some global counters. Information about the following weeks, in particular about the covering requirements, is not available until the solution for the current week has been fixed by the solver.

In the following, a *work stretch* denotes a period of consecutive working days for a nurse. *Rest stretch* and *shift stretch* are analogously defined for periods of consecutive days off and assignments to the same shift, respectively.

There are four hard constraints that have to be fulfilled by any solution to be regarded as feasible:

- **H1. Single assignment per day**: Each nurse can only work a single shift using a single skill per day.
- **H2. Under-staffing**: The minimum number of nurses required for each shift and skill must be present.
- **H3. Shift type successions**: Nurses must not have shifts on two consecutive days that form a forbidden sequence.
- **H4. Missing required skill**: Nurses can only cover assignments for which they have the required skill.

Further, seven soft constraints are defined. Solutions should try to satisfy these constraints, but violating them only results in a penalty to the quality of the solution (weights are listed in the description of each constraint).

- **S1. Insufficient staffing for optimal coverage (30)**: The number of nurses assigned to each shift and skill should not be smaller than the optimum staffing. The penalty is multiplied by the number of missing nurses.
- **S2. Consecutive assignments (15/30)**: The length of each shift stretch (weight 15) and work stretch (weight 30) should be within the bounds defined for the shift type resp. the contract of the involved nurse. The penalty is multiplied by the number of missing or surplus assignments.
- **S3. Consecutive days off (30)**: As before, the length of each rest stretch should be within the bounds defined in each nurse’s contract. The penalty is multiplied by the number of missing or surplus days off.
- **S4. Preferences (10)**: The requests of nurses for shifts (or days) off should be respected.
- **S5. Complete week-end (30)**: Nurses with the complete-weekend constraint in their contract should either work both days of the weekend or none.
- **S6. Total assignments (20)**: Over the whole planning horizon, each nurse’s assignments should be within the bounds defined in their contract.
- **S7. Total working week-ends (30)**: Over the whole planning horizon, each nurse should not work more than the maximum number of weekends defined in their contract.

The complete information necessary to evaluate constraints S6 and S7 is available only after the solution for the last week has been fixed, although they should of course be respected by solvers during all weeks. All sequence constraints (H3, S2, S3) also use the border data from the solution for the previous week (this is provided in the history file).

## Basic model

### Parameters

The first set of parameters contains values that stay the same over the whole planning horizon. These values are stored in the *scenario* file:

*N*:    set of nurses
*S*:    set of shifts
*K*:    set of skills
|*W*|:    number of weeks
$$a^{[+/-]}_n$$
   maximum/minimum assignments for nurse *n* across planning horizon
$$w^{[+/-]}_n$$
   maximum/minimum consecutive working days for nurse *n*
$$f^{[+/-]}_n$$
   maximum/minimum consecutive days off for nurse *n*
$$t^+_n$$
   maximum number of working weekends for nurse *n* across planning horizon
$$b_n$$
   boolean, 1 iff either both days of a weekend should be worked by nurse *n*, or none
$$\kappa _{nk}$$
   boolean, 1 iff nurse *n* has skill *k*
$$\sigma ^{[+/-]}_s$$
   maximum/minimum consecutive assignments of shift *s*
$$u_{st}$$
   boolean, 1 iff shift *t* may be assigned the day after an assignment of shift *s*

The next set of parameters is defined for each *week*.

*w*:    number of the current week
$$c^d_{sk}$$
   minimum cover requirements for day *d*, shift *s* and skill *k*
$$o^d_{sk}$$
   optimum cover requirements for day *d*, shift *s* and skill *k*
$$r^d_{ns}$$
   boolean, 1 iff nurse *n* requested not to work in shift *s* on day *d* ($s=0$ is day-off request)

Finally, these parameters specify values depending on the schedule of the previous week. This *history* is given for the first week and calculated from the solution of the last week for all subsequent weeks.

$$l^{id}_n$$
   id of last shift worked by nurse *n* in previous week (0 if day off)
$$l_{ns}$$
   consecutive shifts of type *s* worked by nurse *n* at the end of the previous week (0 if $s \not = l^{id}_n$)
$$l^{w}_n$$
   consecutive working days for nurse *n* at the end of the previous week (0 if $$l^{id}_n = 0 $$)
$$l^{f}_n$$
   consecutive days off for nurse *n* at the end of the previous week (0 if $l^{id}_n \not = 0$)
$$a^{tot}_n$$
   total number of assignments for nurse *n* so far
$$t^{tot}_n$$
   total number of weekends worked by nurse *n* so far

### Decision variables

$$x^d_{nsk} \in \{0, 1\}$$
$$\forall n \in N, s \in S, k \in K, d \in \{1...7\}$$
$$W_n \in \{0,1\}$$
$$\forall n \in N$$

$x^d_{nsk} = 1$ if nurse *n* is assigned to shift *s* using skill *k* on day *d*, and 0 otherwise.

The $W_n$ variable indicates that nurse *n* works at least one day of the weekend.

The violation of soft constraints is measured using either non-negative or boolean surplus variables:

$$C^{S1}_{skd}$$
   $\ge 0$ missing nurses for optimal coverage of shift *s*, skill *k* on day *d*
$$C^{S2a}_{nsd}$$
   $\ge 0$ missing days in the block of shifts *s* starting on day *d* for nurse *n*
$$C^{S2b}_{nsd}$$
   $\in \{0,1\}$ 1 iff shift *s* of nurse *n* on day *d* violates maximum consecutive shifts
$$C^{S2c}_{nd}$$
   $\ge 0$ missing days in the work block of nurse *n* starting on day *d*
$$C^{S2d}_{nd}$$
   $\in \{0,1\}$ 1 iff work of nurse *n* on day *d* violates maximum consecutive work days
$$C^{S3a}_{nd}$$
   $\ge 0$ missing days in the free block of nurse *n* starting on day *d*
$$C^{S3b}_{nd}$$
   $\in \{0,1\}$ 1 iff day off of nurse *n* on day *d* violates maximum consecutive days off
$$C^{S4}_{nd}$$
   $\in \{0,1\}$ 1 iff assignment on day *d* violates a request of nurse *n*
$$C^{S5}_{n}$$
   $\in \{0,1\}$ 1 iff nurse *n* violates complete weekend constraint
$$C^{S6}_{n}$$
   $\ge 0$ number of total shifts outside the allowed bounds for nurse *n*
$$C^{S7}_{n}$$
   $\ge 0$ number of weekends worked above the maximum by nurse *n*

### Objective function

The objective function is the weighted sum over all violations of each soft constraint:

$$\begin{aligned} minimize ~f =&\,30 * \sum _{\begin{array}{c} s \in S\\ k \in K\\ d \in \{1\ldots 7\} \end{array}} C^{S1}_{skd} \\&+ 15 * \sum _{\begin{array}{c} n \in N\\ s \in S\\ d \in \{1\ldots 7\} \end{array}} (C^{S2a}_{nsd} + C^{S2b}_{nsd}) \\&+ 30 * \sum _{\begin{array}{c} n \in N\\ d \in \{1\ldots 7\} \end{array}} (C^{S2c}_{nd} + C^{S2d}_{nd}) \\&+ 30 * \sum _{\begin{array}{c} n \in N\\ d \in \{1\ldots 7\} \end{array}} (C^{S3a}_{nd} + C^{S3b}_{nd}) \\&+ 10 * \sum _{\begin{array}{c} n \in N\\ d \in \{1\ldots 7\} \end{array}} C^{S4}_{nd} \\&+ 30 * \sum _{n \in N} C^{S5}_n \\&+ 20 * \sum _{n \in N} C^{S6}_n\\&+ 30 * \sum _{n \in N} C^{S7}_n \end{aligned}$$

### Constraints

The following (in)equalities model the hard constraints, as described above.

1 $$\begin{aligned}&\begin{aligned} \text {H1} \quad&\forall n \in N, d \in \{1\ldots 7\} \\&\quad \sum _{\begin{array}{c} s \in S \\ k \in K \end{array}} x^d_{nsk} \le 1 \end{aligned} \end{aligned}$$

2 $$\begin{aligned}&\begin{aligned} \text {H2} \quad&\forall s \in S, k \in K, d \in \{1\ldots 7\} \\&\quad \sum _{n \in N} x^d_{nsk} \ge c^d_{sk} \end{aligned} \end{aligned}$$

For constraint H3, any forbidden shift sequence ($$u_{s_1s_2} = 0 $$) must not be assigned to the same nurse on consecutive days. This must be ensured both within the week (a) and at the boundary of this week with the previous one (i.e. on the first day of the week, b).

3 $$\begin{aligned}&\begin{aligned} \text {H3a} \quad&\forall n \in N, s_1,s_2 \in S, k \in K, d \in \{1 \ldots 6\} : u_{s_1s_2} = 0 \\&\quad \sum _{k \in K} x^d_{ns_1k} + \sum _{k \in K} x^{d+1}_{ns_2k} \le 1 \end{aligned} \end{aligned}$$

4 $$\begin{aligned}&\begin{aligned} \text {H3b} \quad&\forall n \in N, s \in S, k \in K : u_{l^{id}_ns} = 0 \\&\quad x^{1}_{nsk} = 0 \end{aligned} \end{aligned}$$

5 $$\begin{aligned}&\begin{aligned} \text {H4} \quad&\forall n \in N, s \in S, d \in \{1\ldots 7\}, k \in K : \kappa _{nk} = 0 \\&\quad x^d_{nsk} = 0 \end{aligned} \end{aligned}$$

The remaining inequalities deal with the soft constraints. Each inequality can be deactivated by setting the appropriate surplus variable to a value greater than zero, which results in a corresponding penalty in the objective function.

6 $$\begin{aligned} \begin{aligned} \text {S1} \quad&\forall s \in S, k \in K, d \in \{1\ldots 7\} \\&\quad \sum _{n \in N} x^d_{nsk} \ge o^d_{sk} - C^{S1}_{skd} \end{aligned} \end{aligned}$$

S2 actually contains various different constraints that have to be modeled separately: consecutive assignments of the same shift (min (a)/ max (b)) and of work in general (min (c) / max (d)), both during and at the start of the week.

For the minimum consecutive shifts constraints, all patterns that compose a sequence shorter than the required length are prevented. For example, if the minimum number of consecutive night shifts (N) is 4, the patterns {xNx, xNNx, xNNNx}, where x is any other shift or a day off, should not appear.

Since each pattern incurs a penalty proportional to the number of missing assignments, (in the example, xNx would incur a penalty of 45, while xNNNx would incur a penalty of 15) the surplus variables are weighted correspondingly, to ensure that a value of at least the number of missing assignments is necessary to deactivate the constraint.

Equations 8 and 9 model the case where a stretch starts at the beginning of the week or towards the end of the previous week.

7 $$\begin{aligned}&\begin{aligned} \text {S2a} \quad&\forall s \in S, n \in N, b \in \{1\ldots (\sigma ^-_s-1)\}, d \in \{1\ldots 7-(b+1)\} \\&\quad \sum _{k \in K}\left( x^d_{nsk} + \sum _{i \in \{1\ldots b\}} (1-x^{d+i}_{nsk}) + x^{d+b+1}_{nsk}\right) \ge 1 ~-~ \frac{C^{S2a}_{ns(d+1)}}{\sigma ^-_s-b} \end{aligned}\end{aligned}$$

8 $$\begin{aligned}&\begin{aligned} \text {} \quad&\forall s \in S, n \in N, b \in \{1\ldots (\sigma ^-_s-1-l_{ns})\} \\&\quad \sum _{k \in K}\left( \sum _{i \in \{1\ldots b\}}(1- x^i_{nsk}) + x^{b+1}_{nsk}\right) \ge 1 ~-~ \frac{C^{S2a}_{ns1}}{\sigma ^-_s - l_{ns} - b} \end{aligned}\end{aligned}$$

9 $$\begin{aligned}&\begin{aligned} \text {} \quad&\forall s \in S, n \in N : l^{id}_n = s \wedge l_{ns} < \sigma ^-_s \\&\quad \sum _{k \in K}x^{1}_{nsk} \ge 1 ~-~ \frac{C^{S2a}_{ns1}}{\sigma ^-_s - l_{ns}} \end{aligned} \end{aligned}$$

The maximum consecutive shifts constraints is modeled like this: For each shift *s* with a maximum of $\sigma ^+_s$ consecutive assignments, each block of $\sigma ^+_s + 1$ days must contain at least one day where *s* is not assigned. Note that contrary to the situation for S2a, violations of this constraint by more than one shift assignment result in multiple matches of the pattern and therefore it suffices to use boolean surplus variables.

As before, equations 10 model the case where a shift block started in the previous week.

10 $$\begin{aligned}&\begin{aligned} \text {S2b} \quad&\forall s \in S, n \in N, d \in \{1\ldots (7-\sigma ^+_s)\} \\&\quad \sum _{k \in K} \sum _{i \in \{0\ldots \sigma ^+_s\}} x^{d+i}_{nsk} \le \sigma ^+_s ~+~ C^{S2b}_{ns(d+\sigma ^+_s)} \end{aligned} \end{aligned}$$

11 $$\begin{aligned}&\begin{aligned} \text {} \quad&\forall s \in S, n \in N, b \in \{(\sigma ^ +_s - l_{ns} +1)\ldots \sigma ^+_s\} : l^{id}_n = s \\&\quad \sum _{k \in K} \sum _{i \in \{1\ldots b\}} x^i_{nsk} \le b - 1 ~+~ C^{S2b}_{nsb} \end{aligned} \end{aligned}$$

The inequalities modelling the maximum and minimum length of work stretches (S2c, S2d) function analogously to those for shift stretches. The only difference is that an assignment to any shift counts towards the length of the work stretch.

12 $$\begin{aligned}&\begin{aligned} \text {S2c} \quad&\forall n \in N, b \in \{1\ldots (w^-_n-1)\}, d \in \{1\ldots 7-(b+1)\} \\&\quad \sum _{\begin{array}{c} s \in S \\ k \in K \end{array}}\left( x^d_{nsk} + \sum _{i \in \{1\ldots b\}} (1-x^{d+i}_{nsk}) + x^{d+b+1}_{nsk}\right) \ge 1 ~-~ \frac{C^{S2c}_{n(d+1)}}{w^-_n-b} \end{aligned} \end{aligned}$$

13 $$\begin{aligned}&\begin{aligned} \text {} \quad&\forall n \in N, b \in \{1\ldots (w^-_n-1-l^w_{n})\} \\&\quad \sum _{\begin{array}{c} s \in S \\ k \in K \end{array}}\left( \sum _{i \in \{1\ldots b\}} (1-x^i_{nsk}) + x^{b+1}_{nsk}\right) \ge 1 ~-~ \frac{C^{S2c}_{n1}}{w^-_n - l^w_{n} - b} \end{aligned} \end{aligned}$$

14 $$\begin{aligned}&\begin{aligned} \text {} \quad&\forall n \in N : l^{id}_n \not = 0 \wedge l^w_n < w^-_n \\&\quad \sum _{\begin{array}{c} s \in S \\ k \in K \end{array}}x^{1}_{nsk} \ge 1 ~-~ \frac{C^{S2c}_{n1}}{w^-_n - l^w_{n}} \end{aligned} \end{aligned}$$

15 $$\begin{aligned}&\begin{aligned} \text {S2d} \quad&\forall n \in N, d \in \{1\ldots (7-w^+_n)\} \\&\quad \sum _{\begin{array}{c} s \in S \\ k \in K \end{array}} \sum _{i \in \{0\ldots w^+_n\}} x^{d+i}_{nsk} \le w^+_n ~+~ C^{S2d}_{n(d+w^+_n)} \end{aligned} \end{aligned}$$

16 $$\begin{aligned}&\begin{aligned} \text {} \quad&\forall n \in N, b \in \{(w^ +_n - l^w_{n} +1)\ldots w^+_n\} : l^{id}_n \not = 0 \\&\quad \sum _{\begin{array}{c} s \in S \\ k \in K \end{array}} \sum _{i \in \{1\ldots b\}} x^i_{nsk} \le b - 1 ~+~ C^{S2d}_{nb} \end{aligned} \end{aligned}$$

S3 similarily contains two independent constraints: the minimum (a) and maximum (b) number of consecutive days off, again both during and at the start of the week.

The equations modelling these constraints are again analoguous to those from constraints S2c and S2d, except that days of work and days off were swapped.

17 $$\begin{aligned}&\begin{aligned} \text {S3a} \quad&\forall n \in N, b \in \{1\ldots (f^-_n-1)\}, d \in \{1\ldots 7-(b+1)\} \\&\quad \sum _{\begin{array}{c} s \in S \\ k \in K \end{array}}\left( (1 - x^d_{nsk}) + \sum _{i \in \{1\ldots b\}} x^{d+i}_{nsk} +(1- x^{d+b+1}_{nsk})\right) \ge 1 ~-~ \frac{C^{S3a}_{n(d+1)}}{f^-_n-b} \end{aligned} \end{aligned}$$

18 $$\begin{aligned}&\begin{aligned} \text {} \quad&\forall n \in N, b \in \{1\ldots (f^-_n-1-l^f_{n})\} \\&\quad \sum _{\begin{array}{c} s \in S \\ k \in K \end{array}}\left( \sum _{i \in \{1\ldots b\}} x^i_{nsk} - x^{b+1}_{nsk}\right) \ge 0 ~-~ \frac{C^{S3a}_{n1}}{f^-_n - l^f_{n} - b} \end{aligned} \end{aligned}$$

19 $$\begin{aligned}&\begin{aligned} \text {} \quad&\forall n \in N : l^{id}_n = 0 \wedge l^f_n < f^-_n \\&\quad \sum _{\begin{array}{c} s \in S \\ k \in K \end{array}} - x^{1}_{nsk} \ge 0 ~-~ \frac{C^{S3a}_{n1}}{f^-_n - l^f_{n}} \end{aligned} \end{aligned}$$

20 $$\begin{aligned}&\begin{aligned} \text {S3b} \quad&\forall n \in N, d \in \{1\ldots (7-f^+_n)\} \\&\quad \sum _{\begin{array}{c} s \in S \\ k \in K \end{array}} \sum _{i \in \{0\ldots f^+_n\}} x^{d+i}_{nsk} \ge 1~-~ C^{S3b}_{n(d+f^+_n)} \end{aligned} \end{aligned}$$

21 $$\begin{aligned}&\begin{aligned} \text {} \quad&\forall n \in N, b \in \{(f^+_n - l^f_{n} +1)\ldots f^+_n\} : l^{id}_n = 0 \\&\quad \sum _{\begin{array}{c} s \in S \\ k \in K \end{array}} \sum _{i \in \{1\ldots b\}} x^i_{nsk} \ge 1 ~-~ C^{S3b}_{nb} \end{aligned} \end{aligned}$$

To model nurse requests for shifts or days off, any assignment to an unwanted shift incurs the penalty.

22 $$\begin{aligned} \begin{aligned} \text {S4} \quad&\forall n \in N, s \in S, d \in \{1\ldots 7\}: r^d_{ns} \vee r^d_{n0} \\&\quad \sum _{k \in K} x^d_{nsk} \le C^{S4}_{nd} \end{aligned} \end{aligned}$$

For the complete weekends constraint, first the additional helper variables $W_n$ are set if the nurse *n* works either of the days on the weekend. Equations 24 then ensure that if $W_n$ is set, and the complete weekend constraint is present for the nurse, both days of the weekend should have work assigned.

23 $$\begin{aligned}&\begin{aligned} \text {S5} \quad&\forall n \in N, d \in \{6,7\} \\&\quad \sum _{\begin{array}{c} s \in S\\ k \in K \end{array}}x^d_{nsk} \le W_n \end{aligned} \end{aligned}$$

24 $$\begin{aligned}&\begin{aligned} \text {} \quad&\forall n \in N : b_n \\&\quad \sum _{\begin{array}{c} s \in S \\ k \in K \end{array}}(x^6_{nsk} + x^7_{nsk}) \ge 2W_n ~-~ C^{S5}_n \end{aligned} \end{aligned}$$

The constraint S6 (number of total assignments) is modeled slightly differently from the description given by Ceschia et al. (2015). Originally, these constraints were evaluated only after the schedules of all weeks were fixed. In our model, the penalties are calculated immediately and added to the objective function value of the week in which they arise. This does not change the overall quality of the whole schedule, so results are still comparable, although the intermediate quality value of the individual weeks might be different.

25 $$\begin{aligned}&\begin{aligned} \text {S6 } \quad&\forall n \in N \\&\quad \sum _{\begin{array}{c} s \in S \\ k \in K \\ d \in \{1\ldots 7\} \end{array}} x^d_{nsk} \le \max \{a^+_n - a^{tot}_n, ~0\}~+~ C^{S6}_n \end{aligned} \end{aligned}$$

26 $$\begin{aligned}&\begin{aligned} \text {} \quad&\forall n \in N \\&\quad \sum _{\begin{array}{c} s \in S \\ k \in K \\ d \in \{1\ldots 7\} \end{array}} x^d_{nsk} \ge \min \{a^-_n - 7 * (|W| - w), ~7\} ~-~ C^{S6}_n \end{aligned} \end{aligned}$$

The equations for constraint S7 (maximum number of weekends worked) use the variable $W_n$, set in equations 23.

27 $$\begin{aligned}&\begin{aligned} \text {S7} \quad&\forall n \in N \\&\quad t^{tot}_n + W_n \le t^+_n ~+~ C^{S7}_n \end{aligned} \end{aligned}$$

## Model extensions

While the basic model described in Sect. 3 yields feasible solutions that are optimal for each week (if given enough time), the connections between weeks are mostly ignored. Because the weeks are solved individually, solutions are favored that give slightly better results in earlier weeks, at the cost of having potentially much larger penalties in later weeks.

In order to take this into account and improve the overall solution quality, we propose the following extensions to the model, in the form of additional (soft) constraints.

### Overstaffing

**Fig. 1:**
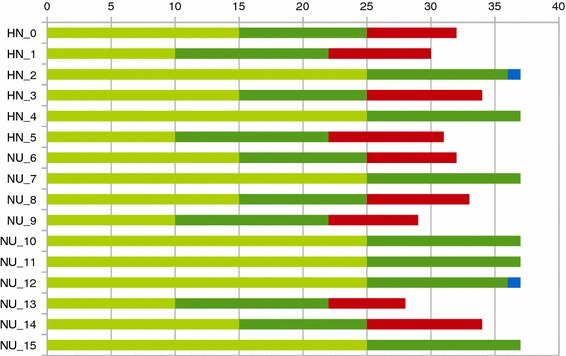
Total number of assignments for the first 15 nurses in the solution of the instance n035w8_2_9-7-2-2-5-7-4-3. *Red* marks assignments exceeding the maximum, *blue* indicates remaining unassigned shifts below the maximum. The *light green* part denotes the minimum number of assignments for each nurse. (Color figure online)

Looking at the total number of shifts for each nurse, one can see that nearly all nurses exceed their maximum number of assignments (compare Fig. 1), except for nurses with a full time contract. Even these nurses mostly have all their available shifts assigned.

This is despite the fact that the total number of assignments is much larger than the amount needed to cover all shifts at the optimal level (1180 assigned versus 1029 needed for optimal staffing levels in the example instance).

However, since there is no penalty on overstaffing, there is no pressure to avoid unnecessary assignments. Indeed, in some cases it can seem advantageous to assign shifts above the optimal staffing levels in order to fulfil sequence constraints or the complete weekend constraints (S7).

However, as soon as the available assignments are used up, high penalties are unavoidable as other constraints (in particular cover constraints and sequence constraints) still have to be fulfilled.

**Fig. 2:**
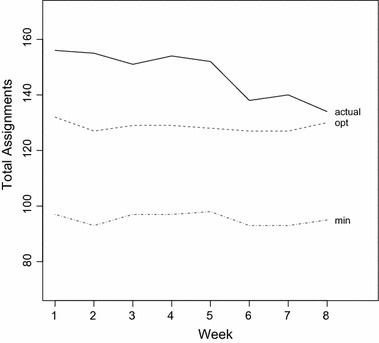
Distribution of the total number of assignments in the eight stages of the instance n035w8_2_9-7-2-2-5-7-4-3

This can also be seen from Fig. 2: In earlier weeks, far more shifts are assigned to nurses than necessary, while in later weeks, constraints S6 force solutions to be closer to the required staffing levels.

To avoid this situation, we introduced a new constraint penalizing overstaffing:

- S8*. Overstaffing: The number of nurses assigned to each shift and skill per day should not exceed the optimal coverage levels.

This constraint can be added to the basic IP model with the following inequalities:

28 $$\begin{aligned} \begin{aligned} \text {S8*} \quad&\forall s \in S, k \in K, d \in \{1\ldots 7\} \\&\quad \sum _{n \in N} x^d_{nsk} \le o^d_{sk} + C^{S8*}_{skd} \end{aligned} \end{aligned}$$

where $C^{S8*}_{skd}$ is a new surplus variable.

### Average assignments

While the overstaffing constraints already reduce the number of excess assignments over the maximum, they do not differentiate between nurses with different contracts. As a result, nurses with part-time or half-time contracts have the same schedules as those with full-time contracts in the earlier weeks. Consequently, they are not available in later weeks without penalties, as their contracts are already maxed out.

Ideally, nurses should be employed according to their contracts during all weeks, with full-time nurses having more assignments per week than other nurses. In part this is already done implicitly, because nurses with shorter contracts usually also have shorter work stretch lengths and longer rest stretch lengths.

To ensure that each nurse will be available until the last stage, their assignments should be distributed evenly across all stages.

- S6*. Average assignments: The total number of assignments up to the current week must be within the bounds defined in the contract, multiplied by the fraction of weeks that have already passed.

This extension generalizes constraints S6 to earlier weeks.

To give an example, if a nurse has a minimum of 10 assignments and a maximum of 22, then after stage 4 (of 8), they should have between 5 and 11 shifts assigned. Assuming that they already had 7 shifts assigned in stages 1–3, this constraint would require them to have between 0 and 4 assignments in stage 4.

If these constraints are satisfied during all weeks, it can be guaranteed that also constraints S6 are satisfied for the whole schedule.

The following inequalities model these constraints:

29 $$\begin{aligned} \begin{aligned} \text {S6*} \quad&\forall n \in N \\&\quad a^{tot}_n + \sum _{\begin{array}{c} s \in S \\ k \in K \\ d \in \{1\ldots 7\} \end{array}} x^d_{nsk} \le \lfloor a^+_n * \frac{w}{|W|} \rfloor ~+~ C^{S6*}_n \end{aligned} \end{aligned}$$

30 $$\begin{aligned} \begin{aligned} \text {} \quad&\forall n \in N \\&\quad a^{tot}_n + \sum _{\begin{array}{c} s \in S \\ k \in K \\ d \in \{1\ldots 7\} \end{array}} x^d_{nsk} \ge \lceil a^-_n * \frac{w}{|W|} \rceil ~-~ C^{S6*}_n \end{aligned} \end{aligned}$$

$C^{S6*}_n$ are again new surplus variables.

Here, fractional limits are rounded such that the limits are always integer numbers and the solutions satisfying S6* always also satisfy S6. We also experimented with different rounding schemes, but this did not influence the quality of the generated solutions.

An alternative version of constraints S6* can be formulated as

- S6*b. Average assignments: In each week, the remaining assignments (not yet used in previous weeks) should be divided equally among all remaining weeks.

Continuing the example above, the nurse in question would have between 3 and 15 assignments left to distribute over 5 weeks (stages 4–8). This means that according to constraint S6*b, they should have between $\frac{3}{5}$ (rounded up to 1) and 3 shifts assigned during stage 4.

31 $$\begin{aligned} \begin{aligned} \text {S6*b} \quad&\forall n \in N \\&\quad \sum _{\begin{array}{c} s \in S \\ k \in K \\ d \in \{1\ldots 7\} \end{array}} x^d_{nsk} \le \lfloor (a^+_n - a^{tot}_n) * \frac{1}{|W| - w + 1} \rfloor ~+~ C^{S6*}_n \end{aligned} \end{aligned}$$

32 $$\begin{aligned} \begin{aligned} \text {} \quad&\forall n \in N \\&\quad \sum _{\begin{array}{c} s \in S \\ k \in K \\ d \in \{1\ldots 7\} \end{array}} x^d_{nsk} \ge \lceil (a^-_n - a^{tot}_n) * \frac{1}{|W| - w + 1} \rceil ~-~ C^{S6*}_n \end{aligned} \end{aligned}$$

The difference between these two formulations becomes visible in case of an imbalance in preceding stages (i.e. too many or too few assignments): S6* tries to restore the balance (which might require unusual work or rest stretches), while S6*b ignores a global balance and works exclusively with the assignments remaining for the current and future stages. A further discussion of these two formulations can be found in Sect. 5.

### Average working weekends

The same argument as above also applies to constraints S7, the maximum number of total weekends. Just like assignments in general, also weekends should not be used up in the early stages, but distributed across all weeks to preserve options.

Therefore, an analoguous constraint S7* can be defined:

- S7*. Average working weekends: In each week, the still available working weekends (not yet used in previous weeks) should be divided equally among all remaining weeks.

with the corresponding inequalities

33 $$\begin{aligned} \begin{aligned} \text {S7*} \quad&\forall n \in N \\&\quad W_n \le \lfloor (t^+_n - t^{tot}_n) * \frac{1}{|W| - w + 1} \rfloor ~+~ C^{S7*}_n \end{aligned} \end{aligned}$$

Note that since there is at most one working weekend per week and nurse, and the maximum number of working weekends is less than the number of weeks, the limit set for each week will either be 0 or 1.

### Next week restrictions

In addition to the global constraints, solutions for different stages influence each other also at the boundary between weeks.

Since the staffing requirements for the next week are unknown in each stage, leaving more options to schedule nurses without conflicts is beneficial. If there are only few good assignments for the nurses with a certain skill, satisfying the cover constraints might become difficult if they do not match one of those options.

**Fig. 3:**
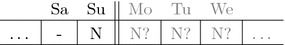
Assignment that heavily restricts options for the following week. Assuming $\sigma ^-_{\text {N}} = 4$, a single night shift on sunday will cause a penalty in the next week if any shifts other than additional night shifts have to be assigned between monday and wednesday

A common way for schedules to restrict the options for the next stage is via the sequence constraints. For example, let the minimum number of consecutive night shifts ($\sigma ^-_{\text {N}}$) be 4 and the proposed solution for this week end with a single night shift on Sunday for a nurse (and any other shift or a day off on Saturday, compare Fig. 3). Then we already know that any assignment for this nurse from Monday to Wednesday that is not a night shift, will inevitably incur a penalty (and depending on the rest of the schedule, assigning only night shifts on these three days could result in penalties of its own).

As another example, if the maximum number of consecutive night shifts is 5 and the proposed solution already contains a shift stretch of at least 5 night shifts in the days leading up to Sunday, this means that assigning a further night shift on Monday of the next week would incur a penalty for exceeding the maximum length.

The same reasoning applies to work and rest stretches.

- S9*. Restriction of next week’s assignments: Options for next week’s schedule should not be restricted. The penalty is calculated as the total number of shifts that cannot be assigned in the next week without violating at least one sequence constraint.

The equations to model this constraint are split into restrictions from shift (a), work (b) and rest (c) stretches, each regarding the minimum and maximum stretch length and with their own set of surplus variables.

Equations 34 detect blocks of the same shift *s* at the end of the week with a length *b* below the minimum shift sequence length (constraint S2a). If such a block is found, this means that for the first $\sigma ^-_s - b$ days of the next week, neither a day off, nor any of the other $|S \setminus \{s\}|$ shifts can be assigned without penalty. In the equation, the left hand side of the inequality sums to $b+1$ in such a case. To still satisfy the constraint, the surplus variable $C^{S9* {}a}_{n}$ must be assigned a value $\ge |S|(\sigma ^-_s-b)$, resulting in a corresponding penalty in the objective function.

The case, where a long shift stretch of shift *s* blocks an assignment of *s* to the first day of the following week, is covered by Eq. 36. Any block of shift *s* at the end of the week with a length of at least $\sigma ^+_s$ violates the corresponding inequality, unless compensated for by a surplus variable.

The restrictions on work (b) and rest (c) stretches work analogously, except that the weights of the surplus variables differ according to the number of blocked assignments.

34 $$\begin{aligned}&\begin{aligned} \text {S9* a} \quad&\forall n \in N, s \in S, b \in \{1 \ldots (\sigma ^-_s-1)\} \\&\quad \sum _{k \in K}\left( (1-x^{7-b}_{nsk}) + \sum _{i \in \{0\ldots (b-1)\}} x^{7-i}_{nsk}\right) \le b + \frac{C^{S9* {}a}_{n}}{|S|(\sigma ^-_s-b)} \end{aligned} \end{aligned}$$

35 $$\begin{aligned}&\begin{aligned} \text {} \quad&\forall n \in N, s \in S \\&\quad \sum _{k \in K}\sum _{i \in \{0\ldots (\sigma ^+_s -1)\}} x^{7-i}_{nsk} \le \sigma ^+_s -1 + C^{S9* {}a}_{n} \end{aligned} \end{aligned}$$

36 $$\begin{aligned}&\begin{aligned} \text {S9* b} \quad&\forall n \in N, b \in \{1 \ldots (w^-_n-1)\} \\&\quad \sum _{\begin{array}{c} s \in S \\ k \in K \end{array}}\left( (1-x^{7-b}_{nsk}) + \sum _{i \in \{0\ldots (b-1)\}} x^{7-i}_{nsk}\right) \le b + \frac{C^{S9* {}b}_n}{w^-_n-b} \end{aligned} \end{aligned}$$

37 $$\begin{aligned}&\begin{aligned} \text {} \quad&\forall n \in N \\&\quad \sum _{\begin{array}{c} s \in S \\ k \in K \end{array}}\sum _{i \in \{0\ldots (w^+_n -1)\}} x^{7-i}_{nsk} \le w^+_n -1 + \frac{C^{S9* {}b}_n}{|S|} \end{aligned} \end{aligned}$$

38 $$\begin{aligned}&\begin{aligned} \text {S9* c} \quad&\forall n \in N, b \in \{1 \ldots (f^-_n-1)\} \\&\quad \sum _{\begin{array}{c} s \in S \\ k \in K \end{array}}\left( x^{7-b}_{nsk} - \sum _{i \in \{0\ldots (b-1)\}} x^{7-i}_{nsk}\right) \le 0 + \frac{C^{S9* {}c}_n}{|S|(w^-_n-b)} \end{aligned} \end{aligned}$$

39 $$\begin{aligned}&\begin{aligned} \text {} \quad&\forall n \in N \\&\quad \sum _{\begin{array}{c} s \in S \\ k \in K \end{array}}\sum _{i \in \{0\ldots (f^+_n -1)\}} x^{7-i}_{nsk} \ge 1 - C^{S9* {}c}_n \end{aligned} \end{aligned}$$

### Unresolvable patterns

**Fig. 4:**
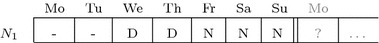
Assuming that $\sigma ^-_\text {N} = 4$ and $w^+_{N_1} = 5$, the maximum work stretch length is already reached but at least one more night shift at the beginning of the next week is required

In the solutions generated for various instances, we found that violations of sequence constraints most commonly appeared at the boundaries between weeks. In many cases, this is the result of patterns similar to those shown in Fig. 4.

In general, not checking the feasibility of completing a multi-shift work stretch in the next week can lead to situations where the last shift stretch can not be extended to the minimum length without violating the maximum work stretch length.

This leads to the following additional constraint:

- S10*. Unresolvable Patterns: For work stretches at the end of the week, there should be a way to complete them in the next week without violating either the maximum work stretch length or the minimum shift stretch length.

Assume a stretch of shift *s* is assigned to nurse *n* at the end of the week. Then an unresolvable pattern has the following structure: First, a block of at least $w^+_n-\sigma ^-_s$ shifts (that can be any type except a day off) is scheduled (A), followed by a single shift that is not *s* (B). Then, the remaining *b* days up to the end of the week are filled with assignments to shift *s* (C), where $b < \sigma ^-_s$.

**Fig. 5:**
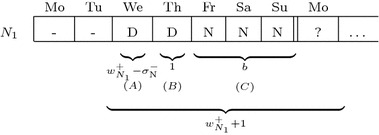
The same pattern as for Fig. 4, split up into the parts matched by the constraints S10*. For this assignment, $b = 3$

To avoid a violation of the minimum shift stretch length, at least $\sigma ^-_s - b$ more days of shift *s* would be required at the start of the next week. However, together with parts (A) and (B), this would bring the total work stretch length to at least $(w^+_n-\sigma ^-_s) + 1 + b + (\sigma ^-_s - b) = w^+_n + 1$, which exceeds the maximum work stretch length $w^+_n$. The different parts are visualized in Fig. 5

Equations 40 detect and penalize these patterns through the use of a further set of surplus variables.

40 $$\begin{aligned} \begin{aligned} \text {S10*} \quad&\forall n \in N, s \in S, b \in \{1\ldots \sigma ^-_s-1\} \\&\quad \sum _{k \in K}( \overbrace{\sum _{\begin{array}{c} j \in \{1\ldots w^+_n-\sigma ^-_s\} \\ t \in S \end{array}} x^{7-b-j}_{ntk}}^{\text {(A)}} + \overbrace{\sum _{t \in S \setminus s} x^{7-b}_{ntk}}^{\text {(B)}} + \overbrace{\sum _{i \in \{0\ldots b-1\}} x^{7-i}_{nsk}}^{\text {(C)}})\\&\qquad \le w^+_n - (\sigma ^-_s - b) + C^{S10*}_n \end{aligned} \end{aligned}$$

### Objective function

To have an impact on the generated solutions, the surplus variables for the added constraints have to be included in the objective function. The objective function $f'$ for the extended model is therefore

$$\begin{aligned} minimize ~f' = \quad f ~+~&W^{S8*} * \sum _{s \in S}\sum _{k \in K}\sum _{d \in \{1\ldots 7\}} C^{S8*}_{skd} \\ ~+~&W^{S6*} * \sum _{n \in N} C^{S6*}_n \\ +~&W^{S7*} * \sum _{n \in N} C^{S7*}_n \\ +~&W^{S9*}* \sum _{n \in N} (C^{S9* a}_n + C^{S9* b}_n + C^{S9* c}_n)\\ +~&W^{S10*} * \sum _{n \in N} C^{S10*}_n \end{aligned}$$

where $W^{S8*}$, $W^{S6*}$, $W^{S7*}$, $W^{S9*}$ and $W^{S10*}$ are the weights of their corresponding constraints.

After a solution has been fixed, the actual penalty has to be recalculated using the objective function of the basic model *f*, to ensure that the penalties from the additional constraints of the extended model are not included in the final result.

Obviously, all constraints introduced in this section should be ignored in the last week, as there is no further week to influence.

## Experimental results

All algorithms were implemented in Java 7, and we used the IBM ILOG CPLEX solver,^2^ version 12.6.3, to solve the IP models. All experiments were performed on an Intel Xeon 2.33GHz PC, using a single thread. The time limit was set to the time alloted by the benchmarking script^3^ provided for the INRC-II (on our machine, between 1 and 9 minutes per stage, depending on the instance size).

We trained the parameters of our models using the parameter-tuning framework irace (López-Ibáñez et al. 2011), over the set of late instances^4^ published for the INRC-II. The models were then evaluated on the set of hidden instances.^5^

### Model extensions

We first evaluated the impact of adding each extension to the basic IP model individually. Due to the similarity in structure and purpose, constraints S6* (Average Assignments) and S7* (Average Weekends) are grouped together. For this comparison, the weight of each additional constraint was set to a value of 1 to ensure that the focus of the optimization still remains on the original constraints. The only exception is constraint S10* (Unresolvable patterns), since a violation of this constraint directly results in a violation of at least one shift stretch length constraint in the next week and thus warrants a weight of 15 (as if the violation had already occured).

**Fig. 6:**
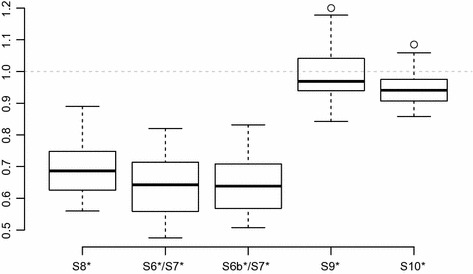
Performance of the basic model extended by each set of constraints individually. The baseline (value of 1) for each instance is the solution generated by the basic model without extensions

Figure 6 shows that each extension improves the quality of the solutions, with the largest impact achieved by S6*/S7* and S8*, those extensions dealing with the two global constraints S6 and S7.

Considering the two variants of S6*, there is next to no difference between the performance of S6* and S6*b.

The solution quality can further be improved by combining multiple extensions. A combination of S6* (and S7*), S8* and S10* produced the best results, each of the extensions further reducing the penalties of the generated solutions. This will be denoted as the *extended model*.

Adding also constraints S9* increased the penalties again, even when S9* was assigned a much lower weight than the other extensions. This is probably connected to the fact that solving models including constraints S9* took nearly twice as long on average and thus optimal solutions could not be found for many stages. However, this cannot be the only reason, as results for models without S9* are also better even for instances where each stage could be solved to optimality.

**Table 1:** Constraint weights for model extensions used for the final evaluations

Param	Weight
$W^{S6*}$	9.9
$W^{S7*}$	9.9
$W^{S8*}$	11.9
$W^{S10*}$	15

**Table 2:** Results for the basic and the extended model over all instances of the hidden dataset

Instance	Basic	Extended	INRC-II		Rank
			Median	Best	
n035w4_0_1-7-1-8	2720	1650	1756.5	1630	3
n035w4_0_4-2-1-6	2625	1950	2021.5	1800	3.1
n035w4_0_5-9-5-6	3020	1775	1928.5	1755	2.2
n035w4_0_9-8-7-7	2700	1680	1723.5	1540	4.3
n035w4_1_0-6-9-2	3035	1755	1737	1500	3.3
n035w4_2_8-6-7-1	2495	1645	1644.5	1490	4.5
n035w4_2_8-8-7-5	2375	1410	1407.5	1255	4.2
n035w4_2_9-2-2-6	2675	1950	1947.5	1705	2.7
n035w4_2_9-7-2-2	2645	2030	1970.5	1650	4.1
n035w4_2_9-9-2-1	2700	1840	1927.5	1620	3.3
n035w8_0_6-2-9-8-7-7-9-8	5640	3550	4171	3020	2.1
n035w8_1_0-8-1-6-1-7-2-0	5380	3360	4045.5	2770	3.3
n035w8_1_0-8-4-0-9-1-3-2	5315	3280	4019	2775	3.7
n035w8_1_1-4-4-9-3-5-3-2	5205	3120	3472.5	2805	4.6
n035w8_1_7-0-6-2-1-1-1-6	5795	3370	3548.5	2840	4.1
n035w8_2_2-1-7-1-8-7-4-2	5570	3390	4205	2910	2.5
n035w8_2_7-1-4-9-2-2-6-7	5725	3445	3699.5	2960	3
n035w8_2_8-8-7-5-0-0-6-9	5265	3250	3603	2815	3
n035w8_2_9-5-6-3-9-9-2-1	6040	3515	3659	3045	2.9
n035w8_2_9-7-2-2-5-7-4-3	5340	3155	3508	2715	3
n070w4_0_3-6-5-1	4580	2775	3151	2700	4.3
n070w4_0_4-9-6-7	4030	2545	2889	2430	2.7
n070w4_0_4-9-7-6	4195	2675	2948	2475	3.8
n070w4_0_8-6-0-8	4440	2850	3016	2435	4.1
n070w4_0_9-1-7-5	4010	2665	2864	2320	4.1
n070w4_1_1-3-8-8	4185	2980	3134.5	2700	3.7
n070w4_2_0-5-6-8	4100	2765	3012	2520	4.1
n070w4_2_3-5-8-2	4250	2800	3141.5	2615	3.5
n070w4_2_5-8-2-5	4460	2820	3005.5	2540	4.1
n070w4_2_9-5-6-5	4315	2820	3046	2615	2.5
n070w8_0_3-3-9-2-3-7-5-2	9690	6065	6222	5115	3.5
n070w8_0_9-3-0-7-2-1-1-0	10,160	6120	6602	5390	3.3
n070w8_1_5-6-8-5-7-8-5-6	9920	6120	6236.5	5475	3.2
n070w8_1_9-8-9-9-2-8-1-4	9715	5740	6018.5	5100	2.9
n070w8_2_4-9-2-0-2-7-0-6	9995	5660	6259	5410	2.9
n070w8_2_5-1-3-0-8-0-5-8	10,310	5810	6315	5280	3.9
n070w8_2_5-7-4-8-7-2-9-9	9885	6010	6317.5	5505	3.9
n070w8_2_6-3-0-1-8-1-5-9	10,785	5590	6255	5120	3.6
n070w8_2_8-6-0-1-6-4-7-8	10,905	5775	6890.5	5350	3
n070w8_2_9-3-5-2-2-9-2-0	10,225	5620	6044.5	5320	2.8
n110w4_0_1-4-2-8	6085	2970	3539	2710	4
n110w4_0_1-9-3-5	6110	3185	3663	2920	2.8
n110w4_1_0-1-6-4	6235	3280	4030	2850	3.9
n110w4_1_0-5-8-8	5930	3125	3569.5	2820	3.3
n110w4_1_2-9-2-0	6810	3810	4092	3345	4
n110w4_1_4-8-7-2	6785	3265	3661	2805	3.9
n110w4_2_0-2-7-0	6170	3610	4198.5	3005	3.5
n110w4_2_5-1-3-0	6650	3240	3637.5	2925	4
n110w4_2_8-9-9-2	6725	3990	4025	3415	4
n110w4_2_9-8-4-9	6265	3415	3769	3135	3.3
n110w8_0_2-1-1-7-2-6-4-7	11,595	5995	6596	5155	3.9
n110w8_0_3-2-4-9-4-1-3-7	12,130	5490	6172.5	4805	4
n110w8_0_5-5-2-2-5-3-4-7	12,015	5570	6227	4750	3.8
n110w8_0_7-8-7-5-9-7-8-1	11,640	5855	6251.5	4855	3.9
n110w8_0_8-8-0-2-3-4-6-3	11,495	5205	6146.5	4465	4
n110w8_0_8-8-2-2-3-2-0-8	12,255	5565	6469	4865	3.4
n110w8_1_0-6-1-0-3-2-9-1	12,010	5895	6514	5090	3.7
n110w8_1_4-1-3-6-8-8-1-3	11,355	5540	6115.5	4315	4
n110w8_2_2-9-5-5-1-8-4-0	12,015	5890	6222.5	4770	4
n110w8_2_8-5-7-3-9-8-8-5	11,465	5570	5809	4360	3.9

Added for comparison are the median and best results achieved by the INRC-II finalists for each instance. The last column contains the average rank among the 7 finalists achieved by our extended IP model for each instance (over 10 runs)

To find optimal weights for the extensions S6*/S7* and S8* (the weight for S10* corresponds directly to the weight of the shifts stretch length constraints), we used IRACE. Both $W^{S6*} (=W^{S7*})$ and $W^{S8*}$ were varied between 0 and 20, with a precision of one significant digit after the decimal point. IRACE was run in parallel on 4 cores with a limit of 5000 iterations.

The best values reported by IRACE are $W^{S6*} = 9.9$ and $W^{S8*} = 11.9$. The two next best configurations are very similar and further experiments showed that the results do not vary significantly under small variations of the weights.

Considering solution times, CPLEX was able to solve most weeks to optimality, even for the larger instances. Over the whole set of hidden instances, using the extended model, an optimal solution could be found for 274 out of 360 weeks. The average gap between the best solution found and the final lower bound for the remaining 86 weeks was only 1.90%, indicating that substantial improvements are not to be expected even with much longer running times.

**Fig. 7:**
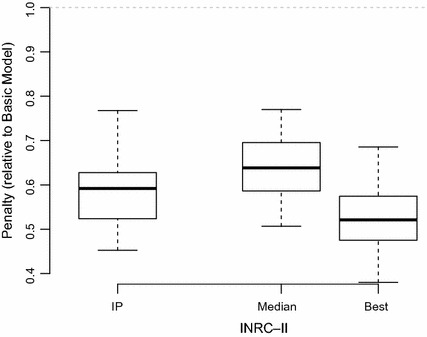
Performance of the extended IP model compared to the solutions produced by the basic model (value of 1). Also shown are the median and best results achieved by the INRC-II finalists

### Final results

For the final evaluation, the extended model was used, with weights for the extensions as shown on Table 1. The exact results can be found on Table 2, see also Fig. 7 for comparison.

Due to the extensions, the penalty incurred by the generated solutions is reduced by about 40% on average, in some cases even to less than half the penalty of the basic model. Further, there is no instance, where the extended model produced results that were not at least 20% better than those of the basic model.

Compared to those of the finalists in the INRC-II, the results of the extended model are competitive (slightly better than the median), although no new best known solutions could be found. The average rank over all instances is 3.45, placing our results firmly into the top half of the finalists.

## Conclusions

In this paper, we have proposed and evaluated several original extensions of standard IP formulations for nurse rostering problems in order to deal with multi-stage settings, as described for the INRC-II.

We have shown that our extensions significantly improve upon the results of the basic model and achieve competitive results compared to the finalists in the competition.

The fact that our model could be solved to (near) optimality in most cases, even under the strict time limits imposed by the challenge, indicates that major improvements cannot be expected from varying solution techniques alone. Instead, future research should be focused on further modifications of the model to distribute the penalties more equally between weeks and avoid blocking options for later weeks. Techniques that try to predict the requirements of yet unknown weeks or distinguish between nurses of different skill sets and contracts could result in even better models.

## Footnotes

1 http://mobiz.vives.be/inrc2/?page_id=226.

2 http://www-03.ibm.com/software/products/en/ibmilogcpleoptistud/.

3 http://mobiz.vives.be/inrc2/?page_id=245.

4 http://mobiz.vives.be/inrc2/wp-content/uploads/2014/08/late-instances.txt.

5 http://mobiz.vives.be/inrc2/wp-content/uploads/2014/08/hidden-instances.txt.
